# The Association between Healthy Diet and Burnout Symptoms among Finnish Municipal Employees

**DOI:** 10.3390/nu13072393

**Published:** 2021-07-13

**Authors:** Markus A. Penttinen, Jenni Virtanen, Marika Laaksonen, Maijaliisa Erkkola, Henna Vepsäläinen, Hannu Kautiainen, Päivi Korhonen

**Affiliations:** 1 Department of General Practice, Turku University and Turku University Hospital, Turku University, 20014 Turku, Finland; jekavi@utu.fi (J.V.); paikor@utu.fi (P.K.); 2 Suomen Terveystalo, 20520 Turku, Finland; 3 Basic Social Security Federation of Municipalities Akseli, 21250 Masku, Finland; 4 Fazer Group, 00941 Helsinki, Finland; marika.laaksonen@fazer.com; 5 Department of Food and Nutrition, University of Helsinki, 00014 Helsinki, Finland; maijaliisa.erkkola@helsinki.fi (M.E.); henna.vepsalainen@helsinki.fi (H.V.); 6 Unit of Primary Health Care, Kuopio University Hospital, 70210 Kuopio, Finland; hannu.kautiainen@medcare.fi; 7 Folkhälsan Research Centre, University of Helsinki, 00290 Helsinki, Finland

**Keywords:** burnout, mental health, nutrition

## Abstract

Background: Burnout is an undesirable mental condition, which may have a negative impact on individuals’ health and work ability. This study aimed to evaluate the relationship between diet and burnout symptoms among female public sector employees. Methods: A cross-sectional study was conducted in 2015 among 630 female employees from 10 municipal work units of the city of Pori, Finland. Burnout symptoms were assessed with the Bergen Burnout Indicator (BBI). The consumption of food items was determined using the food frequency questionnaire (FFQ). The main food groups were categorized into healthy and unhealthy foods based on the Nordic Nutrition Recommendations for a healthy and balanced diet. Results: In multivariate linear regression analysis, consumption of healthy food items had an inverse relationship with the severity of burnout symptoms independently of age, education years, physical activity, and depressive symptoms. De-tailed analysis revealed that subjects with lower BBI score consumed more often low-fat dairy produce, vegetables, fruit and berries, vegetable food, and white meat. Conclusions: Frequent consumption of healthy food items is associated with low level of burnout symptoms. Our results emphasize the importance of diverse and balanced healthy diet to promote work well-being.

## 1. Introduction

There is a growing concern that rapidly changing and demanding work life in industrialized countries can create a risk for mental well-being [1]. Burnout is an unfavorable mental condition resulting, at least partly, from chronic workplace stress that has not been successfully managed [2]. Burnout itself is not a disease, but it is a condition characterized by mood changes (e.g., depressive symptoms, anxiety, stress, and sleep disturbances), which are also present in diagnosed mental diseases (e.g., depression, anxiety disorder) [2]. According to Maslach, burnout symptoms can be separated in three domains: emotional exhaustion, depersonalization, and a sense of reduced accomplishment [2]. Severe burnout may adversely influence work ability leading to absenteeism from work. Therefore, in addition to being a significant threat to individual health, burnout also creates a major economic concern for the society [1,3]. 

There is no established and widely accepted criteria for burnout, but standardized questionnaires are available to evaluate the risk for burnout. The most commonly used questionnaire to examine risk for burnout is Maslach Burnout Inventory (MBI) [4]. In Nordic countries, another questionnaire termed Bergen burnout indicator [BBI-15] is widely used and has been validated in the Finnish population [5]. The evaluation of symptoms of burnout by these questionnaires is a regularly used method in a physical examination at the occupational health to estimate workers mental well-being at work. 

Although several psychosocial risk factors for the development of burnout have been identified, relatively little is known about the role of lifestyle factors in the development of burnout symptoms. Results obtained indicate that regular physical exercise may protect against burnout to some extent [6]. Limited data are available regarding whether diet influences burnout. It has been reported that individuals suffering from burnout may be prone to emotional eating [7], and burnout may be associated with frequent usage of unhealthy food substances [8,9]. Moreover, self-reported healthy eating might be protective against burnout [10]. 

There is growing evidence that balanced diets (e.g., Mediterranean diet and Nordic diet) may play an important role in preventing many chronic diseases including cardiovascular disease, type II diabetes, and obesity [11,12,13,14]. Recently, it has been reported that a healthy diet may also have protective effects against depression [15,16]. Since depression and burnout share similar characteristics, these findings prompted us to study whether diet and various food items are associated with burnout symptoms. We hypothesized that consumption of healthy food items has an inverse relationship with the BBI score.

## 2. Materials and Methods

### 2.1. Participants

The present study is a part of the PORTAAT-study (PORi To Aid Against Threads), which was conducted among municipal employees of the city of Pori in Southwestern Finland in 2014. The study population was derived from ten work units including several professions (e.g., librarians, museum employees, groundkeepers, computer workers, social workers, nurses, physicians, administrative officials, and general office staff). The work units selected had not been involved in any other health promoting program than routine occupational healthcare during the past 10 years. The study population consisted of workers from 10 work units selected by the chief of the welfare unit of Pori. Invitations to the study and information letters were sent by email to the managers of the work units and they further forwarded them to all employees (total number of 2570 workers) by email. In total, 836 employees (33% of those invited; 104 men, 732 women) agreed to participate in the study. For the present analyses, we reported data of the 630 female participants who attended the PORTAAT follow-up study in the year 2015. Only women were included because of the low number of male participants. There were no exclusion criteria for the female participants.

### 2.2. Physical Examination

The participants were invited for clinical examination performed by a trained study nurse in the year 2014. Height was measured with wall-mounted stadiometer and weight with a calibrated scale. Body Mass Index (BMI) was determined as weight [kg] divided by the square of height [m^2^]. Waist circumference was measured midway between the lower rib and iliac crest with the participant in a standing position. For blood pressure (BP) measurements, the participants were in a sitting posture after resting at least 5 min and the results were obtained by using an automatic validated BP monitor. Two readings were performed at intervals of at least 2 min. The mean of these results was used in the analysis.

Age, education, previously diagnosed chronic diseases and the use of regular medication were obtained from questionnaires and patients records. 

Frequency of physical activity (PA) was assessed with a self-administrated questionnaire enquiring for the frequency and duration of leisure-time physical activity (LTPA) and commuting activities in a typical week. Both moderate aerobic PA (e.g., walking) and vigorous PA (e.g., running) were taken into account. Smoking status was obtained with a questionnaire. Non-smoking status was defined as never smoked or quit smoking >12 months ago. Alcohol consumption was determined by using the 3-item Alcohol Use Disorders Identification Test (AUDIT-C) [17]. Sleep quality was assessed with the question “During the past month, how would you rate your sleep quality overall?” (very good, good, poor, or very poor). In the analyses, the two highest classes of sleep quality were combined and set to indicate good sleep quality.

### 2.3. Laboratory Tests

Laboratory tests were determined on blood samples after at least 8 h of fasting (samples were obtained in the year 2015). Plasma glucose, total cholesterol, high-density lipoprotein cholesterol (HDL-C), and triglycerides were detected by enzymatic assay (Architect c4000/c8000). Low-density lipoprotein cholesterol (LDL-C) was calculated by the Friedewald’s formula. 

### 2.4. Psychosocial and Work-Related Factors

The Major Depression Inventory (MDI) was used to reveal depressive symptoms among the study population [18]. MDI is a self-rated questionnaire and it assesses depressive symptoms during the past two weeks. Ten questions are included in the evaluation on a 6-point Likert-type scale from 0 (never) to 5 (all the time) points. The total score ranges from 0 to 50, with a higher score indicating a higher number of depressive symptoms. 

To measure anxiety symptoms, the General Anxiety Disorder 7-item Scale (GAD-7) was used [19]. Each question has a scale from 0 to 3 points and the total score ranges from 0 to 21, with a higher score indicating a higher level of anxiety.

The type of duty (daytime or shift) was obtained from the questionnaire.

The Bergen Burnout Indicator (BBI-15) was used to evaluate the amount of burnout symptoms [5]. The BBI-15 measures three dimensions of burnout: exhaustion, cynicism, and reduced professional efficacy. Fifteen standardized questions are included in the evaluation on a scale from 1 (totally disagree) to 6 (totally agree) points. The total score ranges from 15 to 90. The classification of burnout is as follows: score 15–44 (no burnout), 45–50 (mild burnout), 51–60 (moderate burnout), and 61–90 (severe burnout) [5].

### 2.5. Food Frequency Questionnaire

The participants reported their food consumption frequencies during the past week using a 45-item food frequency questionnaire (FFQ). The FFQ was based on the version developed for and validated among children [20], and with specific attention paid to capture the consumption patterns of vegetables and fruits as well as sugary foods and beverages. A shortened, 25-item version of the FFQ was tested for reproducibility with mostly moderate or good intraclass correlation coefficients [21]. The PORTAAT FFQ included eight food groups: vegetables, fruits, and berries; dairy products; fats and oils; fish; meat and eggs; cereal products; drinks; and others (i.e., sweets and snacks). Additional lines for the frequencies of meals and the use of dietary supplements were included. The FFQ had three answer columns: not at all, times per week, and times per month. The instruction was to either tick the not-at-all column or to write a number in one of the other columns. 

The main food groups in the food frequency questionnaire (FFQ) we categorized into healthy and unhealthy foods (Table 1) based on the previous scientific understanding of the association between diet and cognitive health [15,22] and the Nordic Nutrition Recommendations [23] for the healthy and balanced diet. From the data collected with the FFQ, we summarized the mean daily consumption frequency of foods in the same group and used that as an indicator of dietary consumption in the statistical analysis studying the association between diet and burnout index. For example, the daily consumption frequency of whole grain breads, pasta, rice, porridge, muesli, etc. was summarized together and used as an indicator of whole grain product consumption. Since we did not have the data on energy intake, we adjusted the statistical analysis with physical activity to include the effects of the energy intake in the model. 

### 2.6. Statistical Analyses

The data were presented as means with standard deviations (SD) or as counts with percentages. 

The Bergen Burnout Indicator (BBI) score was divided into quartiles (I ≤ 23, II 24–30, III 31–37 and IV 38–90) (Table 2). Statistical significances for the unadjusted hypothesis of linearity across to quartiles of the BBI score were evaluated by using the Cochran–Armitage test for trend and analysis of variance with an appropriate contrast (linearity). The relationship between BBI quartiles and consumption of healthy and unhealthy food groups consumed per day were evaluated using the Poisson regression models with an appropriate contrast (linearity). Age, education years, leisure-time physical activity, and Body Mass Index were used as covariates in these models. A possible nonlinear relationship between BBI (continuous) and the healthy and unhealthy food items consumed per day were assessed by using 4-knot-restricted cubic spline regression. The length of the distribution of knots was located at the 5th, 35th, 65th, and 95th percentiles. Knot locations are based on Harrell’s recommended percentiles or user-specified points [24]. Multivariate linear regression analysis was used to identify the relationship between BBI as continuous variables and the consumption of healthy and unhealthy food items with standardized regression coefficient Beta [β]. The Beta value was a measure of how strongly the predictor variable influences the criterion variable. The Beta was measured in units of SD. Cohen’s standard for Beta values above 0.10, 0.30, and 0.50 represents small, moderate, and large relationships, respectively. The bootstrap method was used when the theoretical distribution of the test statistics was unknown or in the case of violation of the assumptions (e.g., non-normality). Correlations are expressed with Spearman’s correlation coefficients with bootstrapped 95% confidence intervals. The normality of variables was evaluated graphically and by using the Shapiro–Wilk W test. Stata 16.1 (StataCorp LP; College Station, TX, USA) statistical package was used for the analysis.

### 2.7. Informed Consent and Ethical Approval

Study protocol and consent forms were reviewed and approved by the Ethics Committee of the Hospital District of Southwest Finland. All participants provided written informed consent for the project and subsequent medical research.

## 3. Results

We evaluated 630 female municipal employees with mean age of 49 (SD 10) years (Table 2). Of them 34 (5.4%) had at least moderate symptoms of burnout (BBI score ≥ 51).

Participants with a higher BBI score were slightly more educated, reported less LTPA, poorer quality of sleep, higher level of anxiety, and more depressive symptoms. There were no associations between the groups of BBI score in sociodemographic or lifestyle-related factors or clinical measurements including BMI and waist circumference. Correlation between the MDI and BBI scores was 0.50 [95% CI: 0.44 to 0.56], and between GAD-7 and BBI scores 0.51 [95% CI: 0.45 to 0.57].

Table 3 shows the average consumption of healthy and unhealthy food items. Subjects were divided into groups according to quartiles of the BBI score. Of the healthy food items, participants with lower BBI score consumed more often low-fat dairy produce, vegetables, fruit and berries, vegetable food, and white meat. In contrast, no association in the consumption was observed in seeds and nuts, legume, root vegetables, whole grain products, fish, vegetable oil and margarine, or eggs. None of the unhealthy food items was individually associated with the level of BBI score.

**Figure 1:**
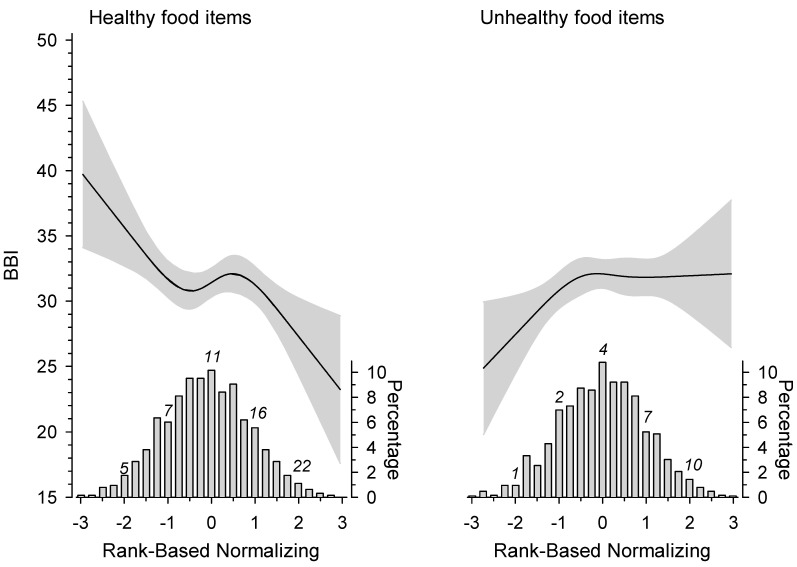
Relationship of the Bergen Burnout Indicator (BBI) score and the distribution of healthy and unhealthy food items consumed per day. The curves were derived from a 4-knot restricted cubic splines regression models. The models were adjusted for age, education years, leisure-time physical activity, smoking, and Body Mass Index. Gray areas represent 95% confidence intervals.

Figure 1 shows the BBI score as a continuous variable according to the distribution of healthy and unhealthy food items´ daily consumption frequency. The more frequent was the consumption of healthy food items, the lower was the BBI score. A linear increase in the BBI score was observed when the subjects consumed unhealthy food items 1–4 times a day, but with higher frequencies the rising BBI score leveled off. 

Correlation coefficient between healthy and unhealthy food items was 0.03 [95% CI: −0.04 to 0.11].

In regression analysis, consumption of healthy food items was found to have an inverse relationship with the BBI score independently of age, education years, LTPA, BMI, and MDI score (Table 4). There was no interaction between healthy and unhealthy diet.

## 4. Discussion

The novel finding of the study is that frequent consumption of certain healthy food items has an inverse negative relationship with the level of burnout. More precise analysis revealed that subjects with lower BBI score consumed more often low-fat dairy produce, vegetables, fruit and berries, vegetable food, and white meat. We also observed that subjects consuming unhealthy food items rarely (maximum three times a day) reported lower BBI scores than women with more frequent consumption. None of the studied unhealthy food items was, however, individually associated with the level of BBI score.

Various psychosocial factors at work leading to long-lasting stressful environment that increase risk for the development of burnout have been identified [25,26]. When burnout is recognized, both individual-directed interventions (e.g., mindfulness techniques, cognitive behavioral techniques) and organizational changes (interventions to reduce the intensity of workload) could be introduced [25,26]. Meta-analyses indicate that there is no proven single efficient intervention available to treat burnout, but in some situations, combined use of these interventions can lead to clinically meaningful reduction in burnout symptoms [25,26]. Thus, further studies are required to reveal the most efficient combinations for specific populations [26]. Therefore, it is of great importance to reveal critical predisposing factors for the generation of burnout symptoms to introduce new efficient intervention options.

In our study, the participants were separated in four groups according to quartiles of the BBI score. As expected, subjects reporting higher BBI score had more symptoms that are evident in burnout (depressive symptoms, anxiety, poor sleeping quality) and, in addition, they practiced less LTPA. In contrast, no associations were observed in various sociodemographic factors (e.g., marital status, shift work), smoking, alcohol consumption, or in any of the clinical factors studied (blood pressure, BMI, waist circumference, lipids, plasma glucose, or usage of medication). These results were unexpected, since many of these factors are important for optimal cardiovascular health (e.g., non-smoking, normal BMI, normal blood pressure, low cholesterol, normal fasting plasma glucose) [27]. Taken together, in our study, most of the studied traditional cardiovascular risk factors did not associate with higher BBI score, suggesting that they do not play a significant role in the generation of burnout symptoms.

Interestingly, we observed strong and linear association between the use of healthy food items and the BBI score. Further analysis revealed that more frequent consumption of certain healthy food items (low-fat dairy produce, vegetables, fruit and berries, vegetable food, and white meat) were associated with lower BBI score. It is noteworthy that among participants consuming no (or rarely) healthy food items the average BBI score was approximately 40. (In BBI-15 the cut-off value for burnout is 45). Thus, although most of these participants do not meet the criteria for burnout, it is likely that they are at increased risk to generate burnout in stressful conditions. To our knowledge, no association between burnout symptoms and diet has been reported. It is clear that additional longitudinal intervention studies are required to elucidate whether consuming healthy food items can prevent burnout in stressful conditions. 

Although our results do not prove a causal relationship between diet and reported burnout symptoms (anxiety, depressive symptoms, and poor sleep quality), it is tempting to speculate possible mechanism(s). Burnout shares several symptoms with depression [15,16]. In recent years, the role of diet in the pathogenesis of mental diseases has been extensively studied. There is evidence that healthy diet can influence the occurrence of mental disorders such as depression [15,16]. The exact mechanism remains to be revealed, but it has been postulated that low-grade inflammation plays a role in the pathogenesis of the disease [16]. Healthy diet has several components known to have anti-inflammatory effects in the body [16,28]. Thus, healthy and balanced diet may have an impact on burnout symptoms by anti-inflammatory mechanism(s). Most likely, several mechanisms are involved since at least in our study, the strongest negative association with the BBI score was observed when all healthy items were regularly and diversely included in the diet. 

Another possibility by which healthy food items may effect mental well-being and protect against burnout symptoms is that food substances affect gut microbiota. It has become evident that gut and central nervous system are tightly linked via a network termed the gut–brain axis that consists of bidirectional communication between the central and the enteric nervous system, linking emotional and cognitive centers of the brain with peripheral intestinal functions [29,30]. Recent findings have revealed the importance of gut microbiota in influencing these interactions, suggesting that gut microbiota can directly affect brain function via these pathways, and thereby affect mood [29,30]. There is evidence that the Western diet has a profound effect in gut microbiota, and consuming daily food items not present in the Western diet (dietary fiber) can modify gut microbiota [31,32]. The disturbance in the balance of gut microbiome (dysbiosis) has been implicated in various diseases common in industrialized countries including diabetes, obesity, inflammatory bowel syndrome, colorectal cancer, and depression [33,34,35]. Thus, it is possible that healthy food items may give growth advantage for the gut bacteria transmitting beneficial signals for brain and thereby leading to positive changes in the mood. 

It is obvious that social and psychological factors influence eating behavior. For example, perceived stress has been reported to be positively associated with the use of several unhealthy food items [36]. Moreover, there is evidence that stressed women are more prone to emotional eating behavior [37]. These results indicate that long-lasting stressful social or psychological environment may lead to the increased consumption of unhealthy food items. Thus, the association between burnout symptoms and dietary behavior may be bidirectional. Interestingly, stress management has been shown to moderate the relationship between stress and the consumption of unhealthy food items [36], indicating that the introduction of education programs to manage stressful situations are likely to help to make good dietary choices. 

There were certain limitations in our study. It was a cross-sectional study and we cannot prove causality of healthy food items protecting against burnout symptoms. Additional intervention studies are needed to address this question. It is possible that burnout leads to unhealthy eating, since it has been reported that individuals with burnout may be prone to emotional eating [7]. In our study, frequent consumption of healthy food items was inversely associated with BBI score and was not influenced by the usage of unhealthy food items. Our study population consisted of female subjects belonging to an active work force, which limits the possibility to draw conclusions that these observations are valid in the general population. It is possible that a healthy worker effect might have an impact on the results [38]. The participation rate was no more than 33%. It is possible that some employees may have ignored the PORTAAT study invitation which was sent by email. Since our study population represents an active work force and the mean scores of MDI and GAD-7 were quite low, it is probable that individuals with major depression or anxiety did not participate in our study. However, we believe that study population was representative, since their mean age and sex ratio were comparable to the entire personnel of the city of Pori [39]. Still, we cannot completely rule out sample selection issues in the design of the study, since males could be underrepresented for non-random reasons. Of note, email surveys generally have about 20% lower response rate than traditional mail surveys [40]. The main results of our study were adjusted with education years, an indirect indicator of socioeconomic status. Nevertheless, individual economic status might have been more indicative of employment characteristics, the ability to maintain healthy habits, and the ability to get help and perhaps mitigate or avoid burnout symptoms. The strength of our study is that the data were collected by using validated questionnaires, and the clinical examination were performed by trained study nurses. We were able to take into account many factors at work and in leisure time. Moreover, this wide-ranging data came from a representative sample of Finnish female municipal employees.

In conclusion, our results show that frequent consumption of healthy food items is associated with a low level of burnout symptoms. Although some of the studied healthy food items were independently inversely associated with BBI score, our results emphasize the importance of diverse and balanced diet.

## Figures and Tables

**Table 1:** Food groups considered healthy or unhealthy.

Healthy Food Groups	Unhealthy Food Groups
Fat-free milk and sour milk, low-fat cheese (fat < 20%)	Red meat, sausages, red cold meat
Unflavored nuts, seeds, and almonds	Juices and beverages sweetened with sugar
Legumes (peas, lentils, beans)	Savory bakery products such as pies and pastries, potato chips and nachos, popcorn, salty nuts
Fresh vegetables	Sweet bakery products (buns, pies, cookies, cakes), chocolate, sweets
Fresh fruits and berries	Alcohol
Whole grain pasta and rice, rye bread, rye crisp bread, breakfast cereal, muesli, porridge	High-fat dairy products: full-fat milk and sour milk, full-fat cheese (fat >20%), butter, butter/oil spreads (fat >80%)
Fish and fish dishes	
Margarines and oils (cooking, bread spread, salad dressing)	
Cooked vegetables	
Eggs	
White meat	

**Table 2:** Characteristics of the study subjects according to quartiles of the Bergen Burnout Indicator (BBI) score.

	Quartiles of BBI Score				P for Linearity
	I [≤23] *n* = 166	II [24–30] *n* = 160	III [31–37] *n* = 148	IV [38–90] *n* = 156	
Sociodemographic factors					
Age, mean [SD]	48.5 [10.4]	48.3 [9.8]	49.2 [9.6]	49.6 [9.5]	0.33
Education years, mean [SD]	13.6 [2.5]	14.2 [2.8]	13.8 [2.6]	14.4 [2.7]	0.023
Cohabiting, *n* [%]	129 [78]	130 [81]	123 [83]	123 [79]	0.49
Shift work, *n* [%]	53 [32]	42 [26]	37 [25]	40 [26]	0.13
Health behaviours					
LTPA, hours per week, mean [SD]	2.9 [2.5]	2.7 [3.3]	2.5 [3.2]	2.2 [2.1]	0.044
Good quality of sleep, *n* [%]	138 [83]	129 [81]	117 [79]	95 [61]	<0.001
AUDIT-C, mean [SD]	2.6 [1.6]	2.7 [1.7]	3.0 [1.6]	2.8 [1.6]	0.11
Smoking, *n* [%]	10 [6]	15 [9]	17 [12]	10 [7]	0.27
Clinical factors					
Blood pressure, mmHg, mean [SD]					
Systolic	132 [18]	130 [17]	129 [16]	132 [18]	0.51
Diastolic	85 [11]	83 [10]	84 [10]	85 [11]	0.96
Body Mass Index, kg/m^2^, mean [SD]	26.6 [4.8]	26.8 [4.6]	26.6 [4.8]	27.0 [5.4]	0.60
Waist, cm, mean [SD]	87.8 [12.3]	88.9 [12.1]	89.0 [13.5]	89.3 [13.5]	0.27
Total cholesterol, mmol/L, mean [SD]	5.20 [0.90]	5.29 [0.93]	5.24 [0.85]	5.34 [0.97]	0.26
LDL cholesterol, mmol/L, mean [SD]	2.94 [0.74]	3.00 [0.75]	2.98 [0.71]	3.02 [0.77]	0.37
HDL cholesterol, mmol/L, mean [SD]	1.78 [0.49]	1.79 [0.46]	1.76 [0.39]	1.82 [0.43]	0.63
Triglycerides, mmol/L, mean [SD]	1.05 [0.47]	1.12 [0.63]	1.11 [0.55]	1.12 [0.62]	0.24
Plasma glucose, mmol/L, mean [SD]	5.50 [0.53]	5.43 [0.49]	5.52 [0.57]	5.52 [0.59]	0.80
Anxiety, GAD-7 score, mean [SD]	1.2 [1.9]	2.1 [2.7]	3.0 [2.8]	5.4 [3.9]	<0.001
Major Depression Inventory, mean [SD]	2.5 [4.2]	3.7 [4.3]	5.3 [4.8]	9.3 [6.9]	<0.001
Regular medication usage					
Antihypertensives	28 [17]	28 [18]	22 [15]	32 [21]	0.65
Statins	6 [4]	5 [3]	12 [8]	7 [4]	0.34

Abbreviations: LTPA, leisure-time physical activity; AUDIT-C, Alcohol Use Disorders Identification Test; LDL, low-density lipoprotein; HDL, high-density lipoprotein; GAD-7, the General Anxiety Disorder 7-item scale.

**Table 3:** Average consumption of healthy and unhealthy food groups consumed per day according to quartiles of the Bergen Burnout Indicator (BBI) score.

	Quartiles of BBI Score				P for Linearity *
	I [≤23] *n* = 166 Mean [SD]	II [24–30] *n* = 160 Mean [SD]	III [31–37] *n* = 148 Mean [SD]	IV [38–90] *n* = 156 Mean [SD]	
Healthy foods	11.99 [0.36]	11.27 [0.34]	11.09 [0.35]	10.63 [0.33]	0.003
Fat-free milk and sour milk, low-fat cheese (fat < 20%)	1.61 [0.12]	1.33 [0.11]	1.42 [0.11]	1.30 [0.11]	0.049
Unflavored nuts, seeds, and almonds	0.37 [0.04]	0.42 [0.05]	0.37 [0.04]	0.33 [0.04]	0.52
Legumes	0.15 [0.02]	0.13 [0.02]	0.17 [0.02]	0.24 [0.03]	0.060
Fresh vegetables	1.61 [0.07]	1.53 [0.07]	1.48 [0.07]	1.41 [0.07]	0.026
Fruits and berries	2.10 [0.10]	1.90 [0.08]	1.89 [0.09]	1.71 [0.08]	0.003
Whole grain products	2.43 [0.12]	2.39 [0.11]	2.26 [0.10]	2.16 [0.09]	0.064
Fish and fish dishes	0.29 [0.02]	0.30 [0.02]	0.29 [0.02]	0.29 [0.02]	0.90
Margarines and oils	1.80 [0.10]	1.76 [0.11]	1.74 [0.11]	1.80 [0.10]	0.62
Cooked vegetables	0.83 [0.05]	0.74 [0.04]	0.76 [0.05]	0.71 [0.04]	0.040
Eggs	0.35 [0.03]	0.32 [0.02]	0.33 [0.02]	0.33 [0.03]	0.72
White meat	0.44 [0.04]	0.45 [0.05]	0.37 [0.03]	0.34 [0.02]	0.049
Unhealthy foods	3.78 [0.19]	3.94 [0.18]	4.21 [0.17]	3.96 [0.17]	0.22
Red meat, sausages, red cold meat	1.25 [0.08]	1.24 [0.06]	1.24 [0.06]	1.23 [0.07]	0.88
Juices and beverages sweetened with sugar	0.10 [0.02]	0.08 [0.02]	0.10 [0.03]	0.16 [0.04]	0.22
Savory bakery products	0.16 [0.02]	0.13 [0.02]	0.18 [0.02]	0.20 [0.02]	0.13
Sweet bakery products	0.92 [0.07]	1.03 [0.08]	1.06 [0.07]	1.00 [0.06]	0.36
Alcohol	0.14 [0.02]	0.14 [0.02]	0.17 [0.02]	0.18 [0.02]	0.19
High-fat dairy products	1.21 [0.11]	1.32 [0.10]	1.46 [0.11]	1.19 [0.10]	0.56

* Adjusted for age, education years, leisure-time physical activity, and Body Mass Index.

**Table 4:** Regression models for the relationship between the Bergen Burnout Index score and consumption of healthy and unhealthy food items.

	Model 1 β [95% CI]	Model 2 β [95% CI]	Model 3 β [95% CI]
Healthy food items	−0.12 [−0.20 to −0.04]	−0.13 [−0.21 to −0.05]	−0.08 [−0.14 to −0.01]
Unhealthy food items	0.07 [−0.00 to 0.16]	0.09 [0.01 to 0.16]	0.06 [−0.01 to 0.13]

Model 1: crude. Model 2: adjusted for age, education years, leisure-time physical activity, and Body Mass Index. Model 3: adjusted for model 2 and Major Depressive Inventory score. Beta coefficients (β) with 95% confidence intervals (CIs).

## Data Availability

The datasets used and/or analyzed during the current study are available from the corresponding author on request.
